# Feasibility study for proton dose calculation of esophageal squamous cell carcinoma based on stopping power ratio directly derived from dual energy CT

**DOI:** 10.3389/fonc.2025.1591139

**Published:** 2025-04-23

**Authors:** Miaomiao Li, Yongbin Cui, Xinjun Lin, Yuanyuan Yan, Qianyu Liu, Mingming Nie, Wenzhen Gong Ye, Yong Huang, Jinhu Chen, Yong Yin

**Affiliations:** ^1^ Shandong University Cancer Center, Shandong University, Jinan, Shandong, China; ^2^ Department of Medical Imaging, Shandong Medical College, Jinan, Shandong, China; ^3^ Department of Radiation Oncology, Shandong Cancer Hospital and Institute, Shandong First Medical University and Shandong Academy of Medical Sciences, Jinan, Shandong, China; ^4^ Department of Radiology, Shandong Cancer Hospital and Institute, Shandong First Medical University and Shandong Academy of Medical Sciences, Jinan, Shandong, China; ^5^ Clinical Science, Philips Healthcare, Beijing, China

**Keywords:** dual-energy CT, proton therapy, photon therapy, stopping power ratio, esophageal squamous cell carcinoma

## Abstract

**Purpose:**

To investigate the feasibility of proton therapy planning using stopping power ratio (SPR) maps directly generated from spectral CT raw data, and to perform a comparative evaluation of dose calculation uncertainties between SPR maps derived from conventional CT Hounsfield Unit (HU) conversion and direct spectral CT SPR generation.

**Materials and methods:**

A retrospective analysis was conducted on 30 patients with mid-thoracic esophageal squamous cell carcinoma (ESCC) who underwent pre-treatment spectral CT imaging. Target volumes and organs at risk (OARs) were delineated on contrast-enhanced CT images and subsequently registered to both non-contrast CT and SPR maps. Three treatment plans were generated: Intensity-modulated radiotherapy (IMRT) plan based on conventional CT, Intensity-modulated proton therapy (IMPT) plan using HU-SPR conversion, IMPT plan utilizing direct SPR maps (IMPT-SPR) from spectral CT. Dose-volume parameters for target volumes and OARs (lungs, heart, spinal cord) were systematically analyzed. Comparative dosimetric analyses were performed among the three plans and between paired groups.

**Results:**

All plans met clinical radiotherapy requirements. For OARs (lungs, heart), IMPT plans demonstrated significantly lower dose-volume parameters compared to IMRT, except for maximum dose (Dmax). Between the two IMPT approaches, no statistically significant differences were observed in dose-volume parameters (*p*>0.05), except for the gradient index which was significantly higher in the HU-converted IMPT plan (*p*<0.05). No significant differences were detected in heart, lung and spinal cord dosimetric parameters between IMPT approaches.

**Conclusion:**

IMPT demonstrated superior OAR sparing compared to IMRT. For mid thoracic ESCC patients under proton therapy, dose calculations based on CT-HU conversion was showed comparable dosimetric impact to DECT-derived SPR in terms of target coverage and OAR protection. These findings support the clinical feasibility of conventional CT-based proton therapy planning and dose calculation.

## Introduction

Esophageal cancer is one of the most prevalent and lethal malignancies worldwide, and radiotherapy plays a crucial role in its treatment (1). Proton therapy, owing to its unique physical property known as the Bragg peak, allows for highly precise dose delivery to the tumor region while significantly reducing radiation exposure to surrounding normal tissues, demonstrating considerable therapeutic potential for esophageal cancer patients (2, 3). However, the accuracy of dose delivery in proton therapy planning depends on the calculation of the stopping power ratio (SPR). The precision of SPR directly determines the proton range, thereby influencing dose calculations. Inaccurate SPR may lead to deviations in treatment dose calculations, potentially compromising therapeutic outcomes or even causing adverse effects (4, 5). Therefore, accurately determining tissue SPR is critical for the design of proton therapy plans and the precision of dose calculations.

Traditional SPR estimation methods rely on empirical conversion models between Hounsfield Unit (HU) values from conventional single-energy CT (SECT) and SPR (6). These methods are based on the assumption of a linear relationship between CT values and SPR values across different tissues. However, due to the complexity of human tissues and the influence of artifacts and noise in CT images, such approaches may introduce significant systematic errors (7–9). In recent years, dual-energy CT (DECT) has emerged as a promising alternative, as it can simultaneously acquire CT images at two different energy levels, enabling more accurate tissue characterization and the generation of electron density maps and effective atomic number (Zeff) maps (10). This capability allows for the direct derivation of SPR maps, offering the potential for more precise SPR estimation compared to conventional single-energy CT (11).

In particular, dual-layer detector DECT, which utilizes two vertically stacked detector layers to simultaneously acquire high- and low-energy data from the same X-ray beam, which significantly improves image acquisition efficiency and consistency (12). Therefore, DECT can produce conventional CT images as well as various quantitative images and parameters. Study conducted at Heidelberg University Hospital in Germany has demonstrated that DECT can reduce the error in SPR estimation from approximately 3.5% to 0.6% in phantom experiments (13). Furthermore, recent research has shown that DECT-based SPR prediction outperforms the clinical standard of single-energy CT in proton therapy planning for prostate cancer patients, providing dose distributions that are closer to the actual values (14).

Although the potential of DECT in SPR estimation has been widely recognized, the clinical application of DECT-derived SPR maps in proton therapy planning for esophageal cancer remains in preliminary research stage (15). Existing studies have primarily focused on SPR estimation for specific anatomical sites, such as head and neck cancer (16, 17). However, for esophageal cancer, which is surrounded by critical organs such as the heart and lungs and influenced by multiple factors, the accuracy of SPR estimation and its comparison with conventional CT-based proton dose calculation methods have not yet been systematically validated (18).

This study aims to evaluate the feasibility of using DECT-derived SPR maps for dose calculation in proton therapy planning for mid-thoracic esophageal cancer and investigate the differences in dose calculations between DECT-derived SPR maps and conventional CT-based indirect SPR conversion methods.

## Materials and methods

### Patients

**Figure 1:**
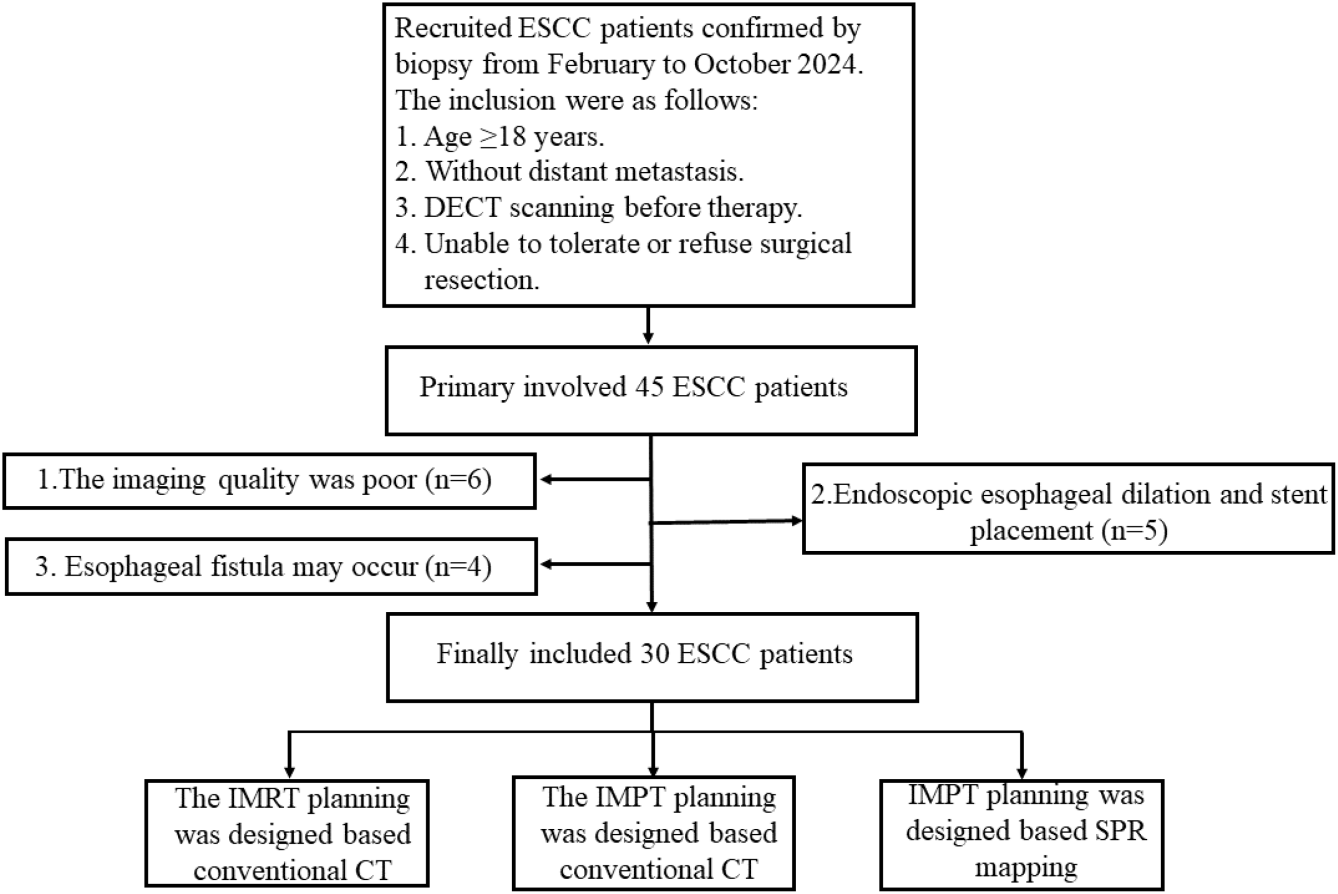
Flow chart of patient inclusion and exclusion criteria.

The retrospective study protocol was approved (No. 2023001010) by our clinical research ethic committee at the Shandong Cancer Hospital and Institute, Shandong First Medical University and Shandong Academy of Medical Sciences. Between February and October 2024, 30 patients with endoscopically confirmed esophageal squamous cell carcinoma (ESCC) were enrolled in our study and classified as stage I (n=5), II (n=3), III (n=22), and IV (n=5) patients according to the 8th edition of the American Joint Committee on Cancer(AJCC) staging manual. The detailed inclusion and exclusion criteria are illustrated in **Figure 1**.

### Scanning parameters and image acquisition

All patients underwent contrast-enhanced CT scans prior to treatment using a spectral CT system (128-slice detector, Spectral CT 7500, Philips Healthcare, Netherlands). Scans were performed in the supine position with the following parameters: tube voltage of 120 kVp, automatic tube current modulation (Dose Right 3D-DOM, Philips Healthcare, Netherlands), pitch of 0.671, tube rotation speed of 0.33 r/s, detector collimation of 128 × 0.625 mm, and a scan matrix of 512 × 512. The CT dose index (CTDIvol) was recorded to monitor radiation dose.

Venous injection of iodinated contrast agent was administered using an automatic injection system (Medrad Stellant CT injector, Bayer Healthcare, Germany). The dose of iodinated contrast agent (Iohexol, Accupaque 350 mg/mL, GE Healthcare, USA) was adjusted according to body weight: 1 mL/kg for patients weighing <55 kg, 100 mL for those weighing 55-120 kg, and 120 mL for those >120 kg. This was followed by an immediate flush with 30 mL of normal saline at an injection rate of 3.0 mL/s. Threshold-triggered CT contrast-enhanced scanning technology was employed. Images were acquired during the arterial, venous, and delayed phases with breath-hold at 30 seconds, 60 seconds, and 90 seconds after the descending aorta reached a CT value of 150 Hounsfield units (HU). The scanning range was determined based on the lesion location and extent but always included the bilateral supraclavicular regions and lungs.

All patients underwent a single scan, and the spectral-based imaging (SBI) data were transferred to the post-processing workstation. Through image reconstruction, conventional CT images, effective atomic number (Zeff) maps, and electron density maps were generated. The stopping power ratio (SPR) was calculated directly using established algorithms based on the Zeff and electron density maps, without relying on the conventional CT-to-SPR conversion. The SPR calculation procedure was based on the Bethe’s equation (19) and Bourque et al. (20) proposed formular.

### Target volume and organs at risk delineation

The delineation of target volumes and OARs as well as dose prescriptions were performed in accordance with international guidelines and the Chinese Society of Clinical Oncology (CSCO) guidelines for the diagnosis and treatment of ESCC. The target volumes were initially delineated by an experienced radiation oncologist based on virtual monochromatic imaging (VMI) at 40 keV from DECT plain scan images, and subsequently copied onto conventional plain CT images.

The gross tumor volume (GTV) consisted of the primary tumor (GTVp) and metastatic lymph nodes (GTVn). GTVp was defined as visible esophageal lesions identified through a combination of imaging modalities (e.g., esophagography, contrast-enhanced CT, MRI, or PET-CT) and endoscopic examinations (e.g., esophagogastroduodenoscopy and/or endoscopic ultrasound). GTVn included visible metastatic lymph nodes, defined as lymph nodes with a short axis diameter ≥10 mm on CT, ultrasound, or MRI (≥5 mm for lymph nodes in the paraesophageal or tracheoesophageal groove regions), or lymph nodes with increased standardized uptake values (SUV) on PET-CT (excluding inflammatory lymph nodes). Additionally, lymph nodes not meeting these criteria but exhibiting specific features, such as significant necrosis, ring-like enhancement, enhancement similar to the primary lesion, or eccentric calcification, were also included in GTVn.

The clinical target volume (CTV) was delineated in accordance with the 2023 Version 2 National Comprehensive Cancer Network (NCCN) Clinical Practice Guidelines for Esophageal and Esophagogastric Junction (EGJ) Cancers. CTVp was defined as a 5–6 mm expansion in the anterior-posterior and lateral directions and a 30 mm expansion in the superior-inferior direction from the GTVp. CTVn was defined as the lymph node regions containing GTVn, with adjustments made to include anatomical barriers as necessary.

The planning target volume (PTV) was generated by expanding the CTV by 5 mm in all directions, with a longitudinal expansion of up to 8 mm, depending on the quality assurance protocols of each center. The delineated target volumes were reviewed and revised by another experienced radiation oncologist. Discrepancies in target volume delineation were resolved through discussions between the two radiation oncologists. OARs, including the lungs, heart, and spinal cord, were automatically delineated using Eclipse V15.5 software. Necessary modifications to the OARs were made by a radiation oncologist based on clinical judgment.

### Treatment plan design

Treatment plans were designed for 30 patients with thoracic ESCC, including intensity-modulated radiotherapy (IMRT), intensity-modulated proton therapy (IMPT), and SPR-based intensity-modulated proton therapy (IMPT-SPR). For photon IMRT plans, treatment planning was performed based on conventional non-contrast CT images with a prescription dose of 50.4 Gy in 28 fractions. Dose constraints for OARs adhered to international guidelines for esophageal cancer radiotherapy. To achieve optimal plan quality, IMRT plans for the 30 patients were designed using 5, 6, or 7 fields, with dose calculations performed using the Acuros XB (AXB) algorithm.

IMPT-SPR plans were designed based on SPR maps generated from DECT images, with dose calculations performed directly on the SPR maps. The prescription dose was 50.4 GyE (relative biological effectiveness, RBE = 1.1). Robust target volumes (RTVs) were generated for each beam to account for uncertainties, with setup uncertainty parameters set to 5 mm and range uncertainty parameters set to 3.5%. IMPT optimization was performed using the NUPO algorithm, and dose calculations were conducted using the PCS algorithm to generate clinically acceptable IMPT plans.

For IMPT plans, the optimization parameters and beam angles from the IMPT-SPR plans were applied to conventional non-contrast CT images. Dose calculations were performed using the HU-to-SPR conversion relationship. The prescription dose was 50.4 GyE (RBE = 1.1), and dose calculations for conventional IMPT plans were also performed using the Monte Carlo (MC) algorithm.

### Analysis of dosimetric parameters

The dosimetric parameters of target volumes and OARs were analyzed based on dose-volume histograms (DVHs) of esophageal cancer patients. For the target volumes, the dose-volume parameters were extracted for each treatment plan, including the dose covering 2% of the target volume (D2), the dose covering 98% of the target volume (D98), and the dose covering 50% of the target volume (D50). Additionally, the homogeneity index (HI) of the target volume was calculated using Equation 1.


(1) $$\;HI=\frac{D_{2}-D_{98}}{D_{50}}$$

The closer the HI is to 0, the more uniform the dose distribution within the target volume. Conversely, a higher HI value indicates a less uniform dose distribution, suggesting the presence of regions with excessively high or low doses within the target volume.

To quantitatively evaluate plan quality, the following volumetric parameters were systematically extracted. VTR: Target volume receiving at least the prescription dose, VT: Total volume receiving the prescription dose, VR: Total target volume. The conformity index (CI) was calculated according to Equation 2, defined as:


(2) $$CI=\frac{V_{TR}}{V_{T}}*\frac{V_{TR}}{V_{R}}$$

where CI values approaching 1.0 indicate optimal dose conformity, with lower values suggesting increased dose spillage beyond the target volume and higher values indicating potential target underdosage.

The target volume receiving 50% of the prescription dose (V50) and the target volume receiving 100% of the prescription dose (V100) were quantitatively assessed. The gradient index (GI) was calculated according to Equation 3, defined as the ratio of V50 to V100 (GI = V50/V100), where lower GI values indicate superior dose fall-off characteristics at the target periphery.


(3) $$GI\;=\;\frac{V_{50}}{V_{100}}$$

A smaller GI indicates a steeper dose fall-off, reflecting a more optimal treatment plan. Conversely, a larger GI value suggests that the high-dose region extends further into normal tissues, potentially increasing their exposure.

The dose-volume parameters for OARs were analyzed. For the heart, the volumes encompassed by the 5 Gy, 10 Gy, 20 Gy, 30 Gy, and 40 Gy isodose lines (V5, V10, V20, V30, V40) were collected, along with the maximum dose (Dmax) and mean dose (Dmean) received by the heart. For the bilateral lungs, the V5, V10, V20, and V30 values, as well as the Dmax and Dmean, were recorded. Corresponding dose-volume parameters were also collected separately for the left lung and right lung. Additionally, the Dmax and Dmean received by the spinal cord were collected.

### Statistical analysis

Normality of data distribution was assessed using the Kolmogorov-Smirnov test. For normally distributed data, intergroup comparisons were performed with analysis of variance (ANOVA) followed by Bonferroni *post hoc* analysis. For non-normally distributed data, the non-parametric Friedman test for related samples and Wilcoxon signed-rank test were employed. All results are expressed as mean ± standard deviation (SD). Statistical analyses were performed using SPSS Statistics version 25.0 (IBM Corp., Armonk, NY, USA). A significance threshold of *p*<0.05 was applied to determine statistical significance.

## Results

### Patient baseline characteristics

**Figure 2:**
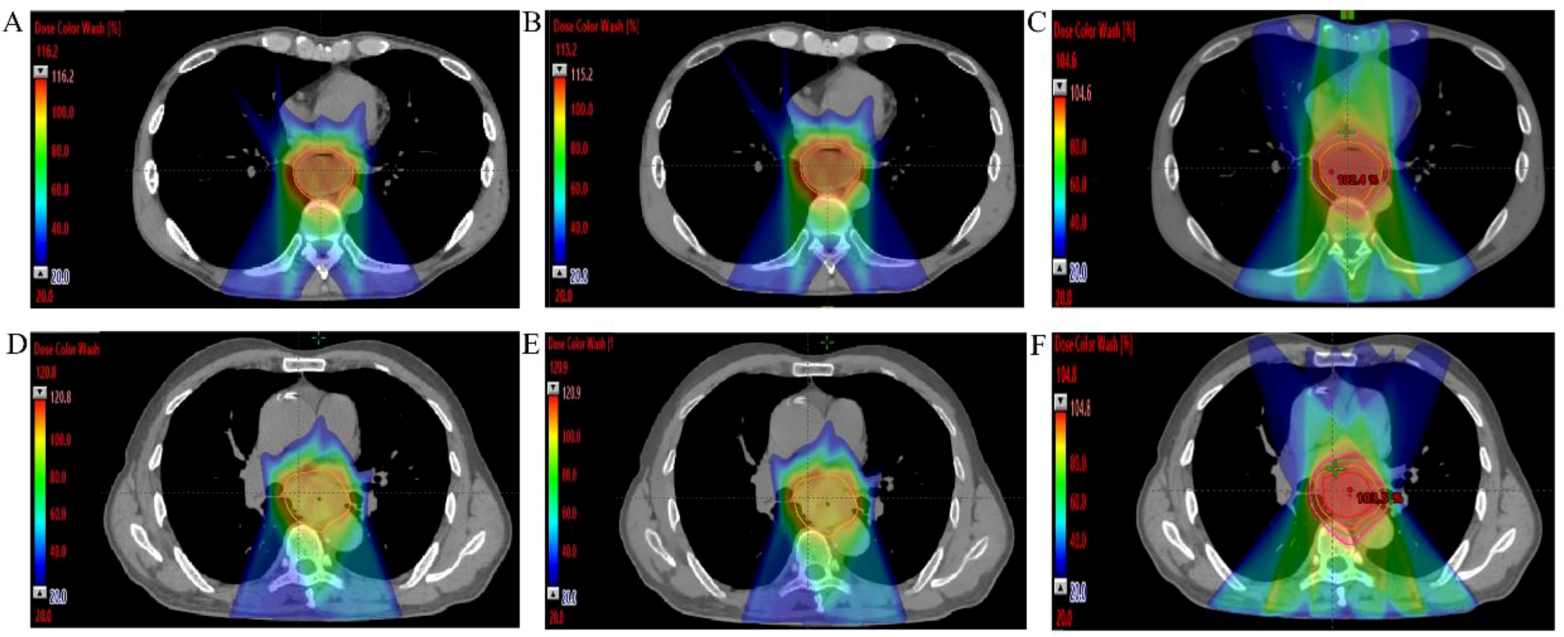
Dose distributions images of three different radiotherapy plans for two mid ESCC patients. **(A–C)** IMPT, IMPT-SPR, and IMRT plans for a 40-year-old male with mid-to-lower thoracic ESCC; **(D–F)** IMPT, IMPT-SPR, and IMRT plans for a 70-year-old male with mid-thoracic ESCC.

This study ultimately included 30 eligible patients with thoracic ESCC, with clinical baseline characteristics summarized in **Table 1**. Each patient had three distinct treatment plans: IMRT, IMPT-SPR, and IMPT plans. The prescribed dose for all plans was 50.4 Gy (Gray equivalent, GyE) delivered in 28 fractions. A total of 90 plans were generated, with at least 95% of the target volume covered by the prescription dose. All plans met clinical requirements for radiotherapy planning. Representative dose distributions of three treatment plans for two patients are illustrated in **Figure 2**.

**Table 1:** Patient baseline characteristics.

Characteristics	Patients	(%)
Age (years)	68 ± 11	
Gender (n)		
Male	24	80%
Female	6	20%
Smoking history (n)		
Yes	16	53%
No	14	47%
Achol history (n)		
Yes	16	53%
No	14	47%
T stage (n)		
T2	4	13%
T3	24	80%
T4	2	7%
N stage (n)		
N0	6	20%
N1-3	24	80%
Tumor location (n)		
upper	3	10%
Middle	20	67%
Lower	7	23%

### Dose-volume parameters for target volumes and organs at risk

**Figure 3:**
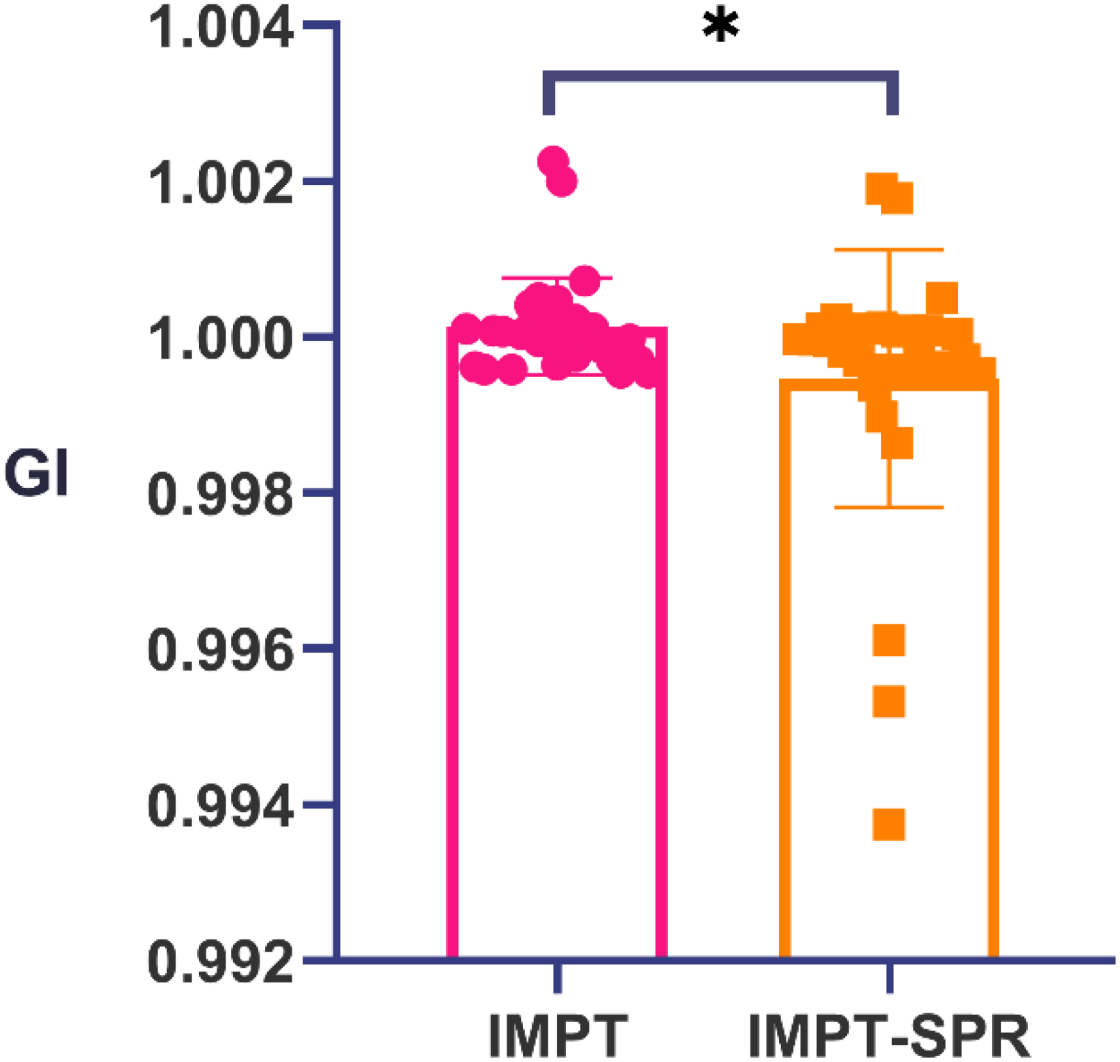
Comparison of gradient index between IMPT and IMPT-SPR plans. * represents p<0.05.

The dose-volume parameters of target volumes and major OARs in the three radiotherapy plans (IMRT, IMPT, and IMPT-SPR) for 30 ESCC patients are summarized in **Table 2**. Plan evaluation was performed using the HI, CI, GI, as well as the Dmean, Dmax, and minimum dose (Dmin) of the target volumes. As shown in **Table 2**, statistically significant differences were observed among the three plans (IMRT, IMPT, and IMPT-SPR) for all parameters except HI and Dmin. *Post hoc* pairwise analysis revealed that, except for HI, the CI, Dmax, and Dmean of the target volumes in the IMRT plan were significantly different from those in the two proton plans (IMPT and IMPT-SPR). In the comparison between the two proton plans, only the GI showed a statistically significant difference between IMPT and IMPT-SPR, as illustrated in **Figure 3**.

**Table 2:** Dose-volume parameters for target volumes and major OARs in the three radiotherapy plans.

Dosimetric parameters	IMPT	IMPT-SPR	IMRT	*P* value	*Post hoc p value*		
					IMPTvs IMPT-SPR	IMPTvs IMRT	IMPT-SPR vs IMRT
CTV/PTV							
HI	0.082 ± 0.019	0.078 ± 0.019	0.076 ± 0.013	0.394	1.000	0.540	1.000
CI	0.525 ± 0.077	0.523 ± 0.076	0.810 ± 0.041	0.000	1.000	0.000	0.000
GI	1.000 ± 0.001	1.000 ± 0.001	1.000 ± 0.000	0.031	0.039	1.000	0.136
Dmax	5901.547 ± 195.992	5878.837 ± 187.263	5544.647 ± 53.557	0.000	1.000	0.000	0.000
Dmin	4390.753 ± 836.694	4416.283 ± 814.583	4152.903 ± 242.416	0.268	1.000	0.553	0.426
Dmean	5318.050 ± 43.427	5316.183 ± 45.224	5285.960 ± 28.228	0.003	1.000	0.007	0.012
Heart							
V5	22.309 ± 9.818	22.963 ± 9.997	76.565 ± 25.042	0.000	1.000	0.000	0.000
V10	17.214 ± 7.788	17.713 ± 7.962	63.319 ± 22.777	0.000	1.000	0.000	0.000
V20	11.227 ± 5.298	11.658 ± 5.459	34.950 ± 13.372	0.000	1.000	0.000	0.000
V30	7.621 ± 3.838	7.978 ± 3.970	17.674 ± 7.172	0.000	1.000	0.000	0.000
V40	4.824 ± 2.634	5.188 ± 2.782	9.286 ± 3.988	0.000	1.000	0.000	0.000
Dmean	576.137 ± 262.660	599.057 ± 271.393	1721.047 ± 548.631	0.000	1.000	0.000	0.000
Dmax	5647.393 ± 467.153	5800.453 ± 282.689	5389.133 ± 75.631	0.000	0.198	0.010	0.000
Bilateral lung							
V5	11.542 ± 5.418	11.797 ± 5.568	40.336 ± 9.860	0.000	1.000	0.000	0.000
V10	8.691 ± 4.283	8.849 ± 4.329	25.945 ± 7.791	0.000	1.000	0.000	0.000
V20	4.554 ± 2.695	4.566 ± 2.691	11.623 ± 4.468	0.000	1.000	0.000	0.000
V30	1.654 ± 1.130	1.636 ± 1.107	5.513 ± 2.881	0.000	1.000	0.000	0.000
Dmean	240.123 ± 117.727	242.317 ± 119.898	787.897 ± 206.114	0.000	1.000	0.000	0.000
Dmax	5842.300 ± 222.946	5789.867 ± 222.766	5446.983 ± 66.431	0.000	0.834	0.000	0.000
Left lung							
V5	13.734 ± 6.598	14.058 ± 6.816	42.657 ± 11.345	0.000	1.000	0.000	0.000
V10	10.232 ± 5.238	10.401 ± 5.347	28.350 ± 8.286	0.000	1.000	0.000	0.000
V20	4.918 ± 3.106	4.921 ± 3.122	13.911 ± 4.955	0.000	1.000	0.000	0.000
V30	1.210 ± 1.079	1.250 ± 1.067	6.468 ± 3.537	0.000	1.000	0.000	0.000
Dmean	264.227 ± 134.102	267.570 ± 136.302	849.230 ± 227.015	0.000	1.000	0.000	0.000
Dmax	5375.057 ± 479.163	5364.783 ± 472.006	5274.923 ± 218.350	0.581	1.000	1.000	1.000
Right lung							
V5	9.979 ± 6.380	10.175 ± 6.554	38.618 ± 10.255	0.000	1.000	0.000	0.000
V10	7.616 ± 5.168	7.713 ± 5.273	24.177 ± 8.869	0.000	1.000	0.000	0.000
V20	4.309 ± 3.205	4.333 ± 3.237	9.919 ± 5.417	0.000	1.000	0.000	0.000
V30	2.014 ± 1.491	1.980 ± 1.481	4.853 ± 3.587	0.000	1.000	0.000	0.000
Dmean	228.143 ± 147.008	226.093 ± 147.772	743.503 ± 232.500	0.000	1.000	0.000	0.000
Dmax	5724.243 ± 392.811	5675.277 ± 405.204	5437.263 ± 77.187	0.002	1.000	0.003	0.019
Spinal cord							
Dmean	950.000 ± 425.376	931.910 ± 433.208	1140.893 ± 495.383	0.146	1.000	0.317	0.231
Dmax	3689.470 ± 334.394	3682.317 ± 341.986	3664.160 ± 165.078	0.902	1.000	1.000	1.000

### Heart dose-volume parameters

In the comparative analysis of heart dosimetric parameters, statistically significant differences were observed in V5, V10, V20, V30, V40, Dmean, and Dmax among the three treatment plans. As demonstrated in **Table 2**, the IMRT plan exhibited significantly higher values in V5, V10, V20, V30, V40, and Dmean compared to both proton therapy plans (IMPT and IMPT-SPR), with all differences reaching statistical significance (*p*<0.05). Notably, the maximum dose to the heart was significantly elevated in both proton therapy plans relative to the IMRT plan. However, no statistically significant differences were detected in heart dose-volume parameters between the two proton therapy approaches.

### Bilateral lung dose-volume parameters

Significant differences were observed among the three treatment plans in terms of V5, V10, V20, V30, and Dmean for the bilateral lungs. Pairwise comparisons showed that the V5, V10, V20, V30, and Dmean values for the bilateral lungs in the IMRT plan were significantly higher than those in the two proton therapy plans (**Table 2**). Conversely, the maximum dose (Dmax) to the bilateral lungs was significantly higher in the two proton therapy plans compared to the IMRT plan, with Dmax values in the IMPT and IMPT-SPR plans exceeding that of the IMRT plan by 395.317 cGy and 342.884 cGy, respectively. No statistically significant differences were observed between the IMPT and IMPT-SPR plans in terms of V5, V10, V20, V30, Dmean, or Dmax for the bilateral lungs.

In the analysis of the left lung, the V5, V10, V20, V30, and Dmean values in the IMRT plan were significantly higher than those in the two proton therapy plans, with all differences reaching statistical significance (**Table 2**). However, no statistically significant differences were observed in the Dmax of the left lung between the IMRT plan and the two proton therapy plans. Similarly, no statistically significant differences were found between the IMPT and IMPT-SPR plans for any dose-volume parameters of the left lung.

For the right lung, the V5, V10, V20, V30, and Dmean values in the IMRT plan were also significantly higher than those in the two proton therapy plans, with statistically significant differences observed (**Table 2**). Conversely, the Dmax of the right lung was significantly higher in the two proton therapy plans compared to the IMRT plan, with Dmax values in the IMPT and IMPT-SPR plans exceeding that of the IMRT plan by 286.98 cGy and 238.014 cGy, respectively. No statistically significant differences were observed between the IMPT and IMPT-SPR plans for any dose-volume parameters of the right lung.

### Spinal cord dose parameters

**Figure 4:**
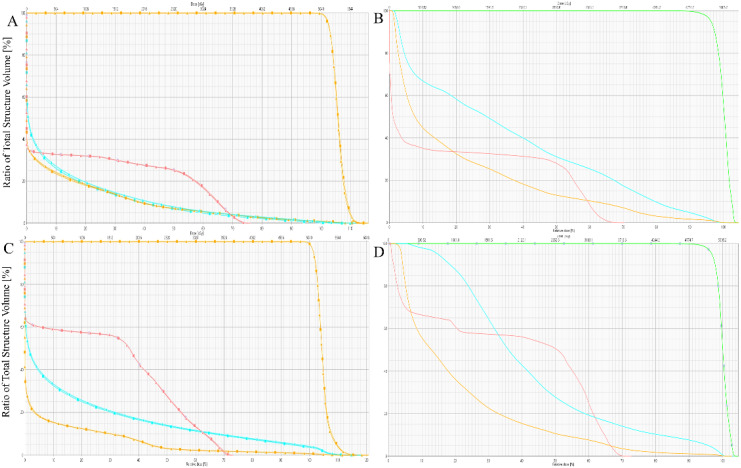
Comparison of DVHs for IMPT, IMPT-SPR, and IMRT Plans in Two Patients. **(A, B)** represent one patient, and **(C, D)** represent another. In the DVHs, the triangular line represents the IMPT plan, the square line represents the IMPT-SPR plan, and the solid line represents the IMRT plan. Red indicates the spinal cord, blue indicates the heart, and orange indicates the bilateral lungs.

The Dmax and Dmean to the spinal cord demonstrated no statistically significant differences among the three treatment plans (*p*>0.05). However, the photon-based IMRT plan exhibited significantly higher Dmean values compared to both proton therapy approaches (IMPT and IMPT-SPR), with mean dose elevations of 190.853 cGy (IMRT vs IMPT) and 208.983 cGy (IMRT vs IMPT-SPR), respectively. Quantitative analysis revealed comparable maximum dose levels across all plans: 3689.470 ± 334.394 cGy (IMPT), 3682.317 ± 341.986 cGy (IMPT-SPR), and 3664.160 ± 165.078 cGy (IMRT), with no statistically significant inter-group variations (*p*=0.902). **Figure 4** provides comparative DVH analyses from two representative cases, demonstrating the differential dose distributions among IMPT, IMPT-SPR, and IMRT treatment plans.

## Discussion

Current clinical validation of SPR-based dose calculations predominantly focuses on head and neck or pelvic regions, with limited research on the feasibility and phantom dose verification of SPR-based proton therapy planning for esophageal cancer (14, 21). This study evaluates the feasibility of using DECT-derived SPR maps for dose calculation in proton therapy for mid esophageal cancer. By comparing IMRT plans, conventional CT-based IMPT plans, and IMPT-SPR plans, we demonstrate the significant dosimetric advantages of proton therapy over photon therapy in sparing OARs. While confirming that the uncertainties associated with conventional HU-based SPR conversion for IMPT planning and dose calculation are clinically acceptable for the mid ESCC patients. However, in the clinical workflow, as for the patients with long target or highly complex treatment plans may particularly benefit from DECT-derived SPR-based planning.

In the target coverage dose-volume parameters comparison, IMPT plans exhibited higher mean and maximum doses compared to IMRT plans, consistent with previous findings (22), suggesting that proton therapy may offer superior tumor control (23). The CI of proton plans was lower than that of photon plans, as IMPT planning was based on the CTV and optimized using the RTV instead of the PTV, eliminating the need to evaluate PTV coverage. Although the GI showed statistically significant differences among the three planning methods, the values were close, with both proton and photon plans approaching 1.

In the dosimetric analysis of OARs, our results indicate that proton therapy plans (including conventional IMPT and SPR-based IMPT) significantly reduced the mean dose and dose-volume parameters (V5, V10, V20, V30, V40) for the heart, lungs, and spinal cord (24). This aligns with the known dosimetric benefits of proton therapy, attributed to the Bragg peak effect, which enables superior target coverage while minimizing dose to surrounding normal tissues (25). This advantage is particularly critical for esophageal cancer due to the proximity of critical structures such as the heart and lungs, which increases the risk of radiation-induced complications. However, the maximum doses to the heart and lungs (especially the right lung) were higher in proton plans compared to IMRT, likely due to the mid-thoracic location of the esophageal tumors in our cohort (26). This highlights that while IMPT is a promising modality for minimizing radiation-induced toxicity while maintaining effective tumor control, attention should be paid to the maximum doses delivered to normal tissues.

Comparison between conventional IMPT and IMPT-SPR plans, no statistically significant differences were observed in the heart’s V5, V10, V20, V30, V40, Dmean and Dmax. This suggests that, for mid-thoracic esophageal cancer, the dosimetric uncertainties associated with conventional CT-based proton planning and dose calculation are clinically acceptable from the perspective of heart sparing. Similarly, no significant differences were found in the dose-volume parameters (V5, V10, V20, V30, V40, Dmean, Dmax) for the lungs between conventional IMPT and SPR-based IMPT plans, whether analyzed collectively or separately (left and right lungs). This further supports the clinical acceptability of conventional CT-based proton planning for lung sparing in most mid-thoracic esophageal cancer cases. Additionally, no significant differences were observed in the mean or maximum doses to the spinal cord between conventional IMPT and SPR-based IMPT plans, reinforcing the feasibility of conventional CT-based proton planning. This may be attributed to the established range uncertainty parameter of 3%-3.5% (4, 27), which generally meets clinical requirements for most mid-thoracic esophageal cancer cases when respiratory motion is not considered (28–30).

Our study has several limitations. First, this study focuses exclusively on mid-thoracic esophageal cancer patients with smaller lesions and lower planning complexity, which may not be generalizable to cases with longer target volumes or higher planning complexity. Second, the single-center retrospective is designed with a sample size of 30 patients, which limits the generalizability of our findings, necessitating larger, multi-institutional studies to validate the applicability of conventional CT-based IMPT planning. Third, this study is a preliminary radiotherapy plan feasible and comparative study, which absences the end-to-end validation using phantoms and actual long-term treatment outcomes and toxicity reactions of patients (5, 31). Last, the advanced motion management techniques (e.g., 4D-CT simulation, respiratory gating) were necessary to be applied for investigation the uncertainties in dose distribution caused by respiratory motion.

## Conclusions

Our study highlights the dosimetric advantages of IMPT over IMRT in OARs, such as the heart, lungs, and spinal cord, in esophageal cancer patients (*p*<0.05). Additionally, we evaluated the uncertainties associated with dose calculations based on SPR values derived from HU values obtained from conventional CT images. Our findings demonstrate that, in the absence of spectral CT, the dose calculation errors for IMPT plans based on conventional CT images are within an acceptable range for patients with mid-thoracic esophageal cancer.

## Data Availability

The raw data supporting the conclusions of this article will be made available by the authors, without undue reservation.
